# Vector characteristics of sub-ps 1.9 µm pulses produced by non-PM thulium-doped all-fiber MOPA

**DOI:** 10.1038/s41598-026-58551-6

**Published:** 2026-06-20

**Authors:** Daniil Batov, Vasilii Voropaev, Semyon Mizgirev, Vladimir Lazarev, Mikhail Tarabrin

**Affiliations:** Science and Education Center for Photonics and IR-Technology, Bauman Moscow State Technical University, Moscow, Russia 105005

**Keywords:** Optics and photonics, Physics

## Abstract

The efficiency of using 1.9 µm ultrashort pulses generated by thulium-doped all-fiber master oscillator power amplifiers (MOPAs) in various applications, such as material ablation, supercontinuum and Raman soliton generation, and other fields, depends not only on the amplitude-phase characteristics but also on the polarization state of the pulse. Despite the advantages of non-polarization-maintaining (non-PM) MOPAs, such as ease of assembly, low cost of fibers, and greater flexibility of parameters, the main disadvantages are sensitivity to external influences and the possibility of generating pulses with complex polarization. Thus, non-PM MOPAs have the potential to be used in laboratory settings, and tuning the pulse electric field vector parameters can improve their application efficiency. However, pulse electric field vector measurements have not been widely used in laser development research due to their complexity. Therefore, the objective of this study is to perform vector electric field measurements of sub-ps pulses at a wavelength of 1.9 µm generated by a non-PM thulium-doped all-fiber MOPA. This study presents vector measurements of the pulses at different polarization controller settings and average radiation power levels of the developed MOPA by using the tomographic ultrafast reconstruction of transverse light E-fields (TURTLE) principle, highlighting the importance of such measurements.

## Introduction

Ultrashort pulses at a wavelength of 1.9 µm have a wide range of potential applications such as supercontinuum^1–5^ and Raman soliton generation in the mid-IR range^6–9^, material processing^10–13^, surgery^14^, study of nonlinear characteristics of various materials^15–17^, nonlinear microscopy^18,19^, and others. In all the indicated areas of application, the amplitude-phase characteristics, as well as the polarization of the pulses, are of great importance, since they determine the interaction of radiation with the medium. During micro-modification of the surface with femtosecond pulses, it was shown that the type of pulse polarization influences both the ablation efficiency and the shape of the resulting craters^11^. Paper^12^ shows that using elliptically polarized light increases the efficiency by a factor of 2 compared to linearly polarized light for writing birefringent voxels in silica glass that can be used for data storage. When generating supercontinuum and Raman solitons, the polarization of the radiation also influences the interaction process, so for linear polarization, the nonlinear refractive index is 50% greater than for circular polarization^20^, which can influence the efficiency of generation. An increase in the ablation efficiency of both inorganic and biological targets is demonstrated to be inversely proportional to the square of the ultrashort pulse duration at constant energy^21^. The quality of the pulse shape and peak power affects the fluorescence intensity in two-photon microscopy^22^. In a study of resonance phenomena^23^, a change in the trajectories and distributions of electrons caused by the presence of a pedestal at the leading edge of an ultrashort laser pulse was demonstrated.

To achieve high-intensity ultrashort pulses at a wavelength of 1.9 µm, a thulium-doped all-fiber master oscillator power amplifier (MOPA) can be used^24–30^. At the same time, non-polarization-maintaining (non-PM) laser systems have lower-cost fiber manufacturing technology and simpler assembly (no need to align fiber axes during splicing) compared to polarization-maintaining (PM) schemes. The price of PM fibers can be 2.5–10 times higher than that of standard fibers. Splicing PM fibers requires a special splicing machine that aligns the slow and fast axes of the PM fibers. This machine is 5–10 times more expensive than a standard splicing machine. Moreover, when splicing some dispersion-compensation PM fibers with conventional PM fibers, polarization is poorly preserved, and the polarization extinction ratio (PER) drops to 10 dB^31^. To assemble a PM thulium-doped laser operating in the dispersion-managed soliton regime with a broadband spectrum and high PER, it is necessary to use specially developed experimental fibers that are not yet commercially available^32^. However, the main drawback of non-PM designs is the influence of vibrations, temperature, and humidity changes on the output regime, requiring periodic tuning of the output regime using polarization controllers (PCs). In addition, non-PM systems can generate pulses with complex polarization over time, which is caused by the birefringence of various components of the laser system (isolators, fibers with random birefringence, PCs, and others) and the effect of nonlinear polarization rotation^33,34^, although the influence of these effects is quite difficult to predict^35^. Thus, non-PM thulium-doped all-fiber MOPAs are suitable and widely used sources for conducting studies in stable conditions, such as laboratories, but for effective use, they require tuning of the generation regime and the electric field vector along the pulse due to the unpredictability of its polarization and amplitude-phase characteristics.

To adjust the electric field vector of the generated pulse, it is necessary to measure it. Single-scan methods such as POLarized Light Interference versus Wavelength of Only a Glint (POLLIWOG)^36^ and vectorial frequency-resolved optical gating (V-FROG)^37^ and multiple-scan methods such as tomographic ultrafast reconstruction of transverse light E-fields (TURTLE)^38^, D-TURTLE (Dispersion-scan TURTLE)^39^ can be used to measure the electric field vector of an ultrashort pulse. At the same time, measurements of the pulse electric field vector, which include the polarization state and amplitude-phase characteristics, are not widely used in scientific papers devoted to ultrafast fiber laser development due to their complexity. The overwhelming majority of studies measure the autocorrelation function of a single polarization projection of the pulse, while fewer studies present measurements of the amplitude-phase characteristics of the pulses. Measurements of the electric field vector of the developed fiber lasers were only performed in this work^40^ and in our previous work^35^, to the best of our knowledge.

Thus, the objective of this work is to experimentally demonstrate the measurement of the electric field vector of sub-ps pulses at a wavelength of 1.9 µm generated by a non-PM thulium-doped all-fiber MOPA. As a source of pulses with complex polarization and amplitude-phase characteristics, we use a non-PM thulium-doped all-fiber system we developed earlier with a minimum pulse duration in one polarization projection at the level of 70 fs^29^. To measure the electric field vector of pulses, the TURTLE method is used, which is based on measurements of the power and amplitude-phase characteristics of three polarization projections of pulses, two of which are orthogonal^38^. The amplitude-phase characteristics of the pulses are measured by the second harmonic generation (SHG) FROG method^41^. Thus, the electric field vector characteristics are obtained from three SHG-FROG traces measured at different linear polarizer angles. The paper presents a sequential description of the experimental setup and measurements of the electric field vector of pulses at various settings of the laser system PCs and different output radiation powers and demonstrates the importance and features of the presented measurements.

## Experimental setup

**Fig. 1:**
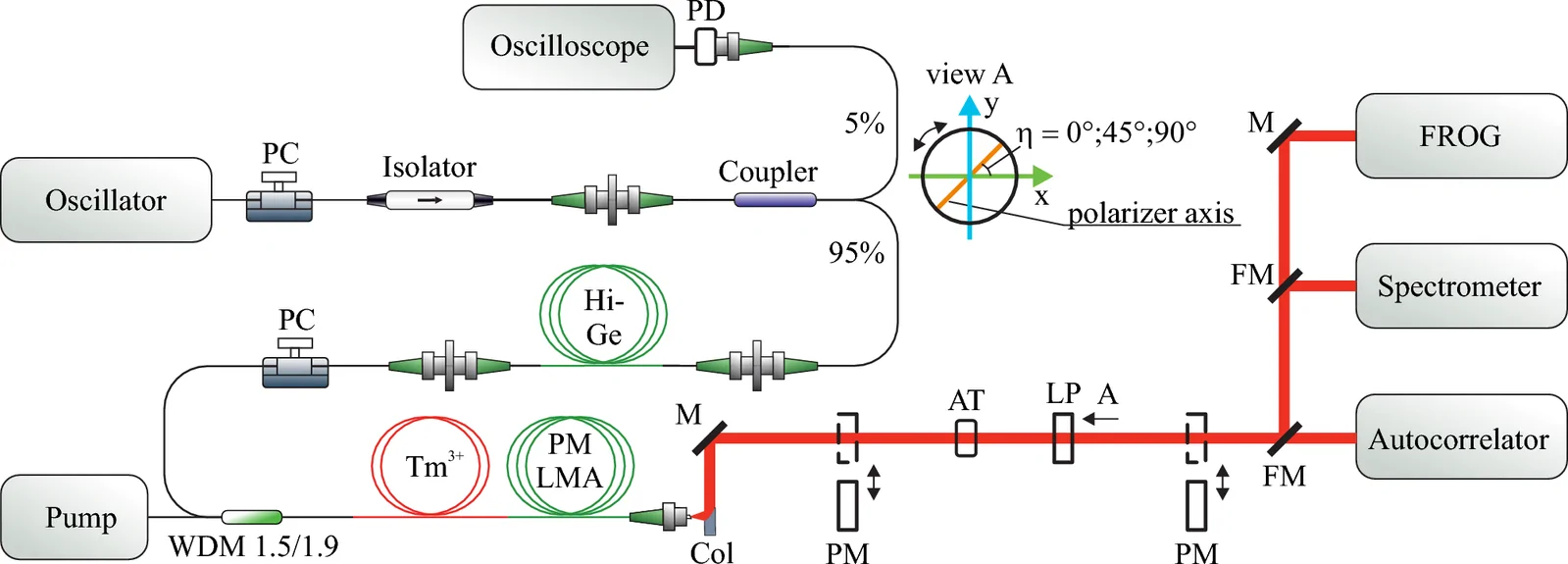
Schematic of experimental setup: *PD* photodetector; *Hi-Ge* fiber with high concentration of germanium oxide for pulse stretching; *PC* polarization controller; *WDM* wavelength division multiplexer; *Tm*$^{3+}$ thulium-doped fiber; *PM LMA* polarization-maintaining large mode area fiber; *Col* reflective collimator; *AT* attenuator; *PM* power meter; *LP* linear polarizer; *M* mirror; *FM* flipping mirror.

A schematic diagram of the experimental setup, including the previously developed MOPA^29,42^, is shown in Fig. 1. The output pulses of the hybrid mode-locked thulium-doped fiber oscillator have a repetition rate of 24 MHz, a spectral width of about 20 nm, a central wavelength of around 1.9 µm, a pulse duration of 330 fs, and an average power of about 5 mW^35,42^. A polarization-independent isolator is used in the scheme to prevent pulses from being reflected back into the oscillator. As shown in the work^35^, for different settings of the PC at the laser output, pulses passing through a polarization-independent isolator due to the action of birefringence can either be split into two pulses with elliptical orthogonal polarization with a delay between them of about 500 fs or not be split into two and have a polarization close to linear and low-energy sub-pulses.

To control the mode-locking regime, the radiation with 5% of power is directed using a fused fiber coupler to a 10 GHz PIN photodetector (EOT-5000F, Coherent, USA) connected to a 1 GHz oscilloscope (104MXs, LeCroy, USA). The type of pulse at the isolator output was monitored by connecting the FC/APC connector with 5% radiation power to the collimator (Col) and measuring the spectra for different polarizer (LP) angles. For double pulses, the spectrum had two peaks, and the intensity ratio and the position of the peaks varied depending on the polarizer angle. For single pulses, rotating the polarizer resulted in a single peak, the intensity of which varied depending on the polarizer angle.

Then pulses pass through a Hi-Ge fiber with a high concentration of germanium oxide in the core and normal group velocity dispersion (GVD) to increase the pulse duration during amplification. The stretched pulses are amplified in a thulium-doped fiber with normal GVD, which significantly broadens the spectrum and pulse duration during amplification. Such pulse dynamics are manifested due to the simultaneous action of dispersion broadening, self-phase modulation, and amplification. The thulium-doped fiber is pumped by an erbium-ytterbium fiber laser at a wavelength of 1.55 µm. A wavelength division multiplexer (WDM 1.5/1.9) is used to couple the pump radiation into the active fiber. After the active fiber, the pulses enter a PM LMA fiber with a large mode area for pulse compression. The PM LMA fiber is a highly birefringent panda-type fiber. Its ability to modify the pulse polarization contributes to the formation of a complex intra-pulse vector structure, which is useful for demonstrating the vector-field measurement. A PC installed before the WDM is used to change the polarization of the pulses. All fibers except PM LMA are not highly birefringent. The maximum average output power of the amplifier is about 1 W. A more detailed description of the amplifier used is given in paper^29^.

To output pulses from the amplifier, a reflective collimator (Col, RC02APC-P01, Thorlabs, USA) is used. All mirrors used have a metal coating that does not change the polarization state. The TURTLE method is used to measure the electric field vector of pulses. This method involves measuring the average power and amplitude-phase characteristics of pulses in three polarization projections, two of which must be orthogonal, with the third projection typically at an angle of 45$^{\circ }$ to the orthogonal one. Using the measured data and specialized mathematical apparatus, the relative delay (*τ*) and phase (*θ*) between the amplitude-phase characteristics of pulses in two orthogonal projections are found. Knowledge of the *τ*, *θ* and the amplitude-phase characteristics of orthogonal polarizations of the pulse almost completely determines the electric field vector of the pulse, with the exception of the absolute phase and time direction. The uncertainty in the time direction is not important in this study but can be easily eliminated using an additional measurement introducing a known chirp into the pulse^38^. A detailed description of the mathematical apparatus for TURTLE retrieval was presented previously^35,38^, while a more detailed description of the measurement method is provided in Appendix A. To measure the amplitude-phase characteristics of the pulse, we use a homemade FROG setup^43^, based on non-collinear SHG in a BBO crystal. The SHG-FROG setup has two measurement range limiting factors. The first is the delay line, which determines the range of measured delays and the delay resolution. The second factor is the nonlinear crystal and spectrometer, which determine the spectral range and spectral resolution. In our setup, the measured delay range is 80 ps with a step size of 0.3 fs. The spectral range of the pulses that satisfy the phase matching condition in the nonlinear crystal is 200 nm, and the maximum resolution of the spectrometer is 0.02 nm. For a linearly polarized Gaussian pulse, this corresponds to a measured duration range of 42 fs to 17 ps, provided that the spectral width at half-maximum is no greater than 126 nm and no less than 0.5 nm. To isolate the polarization projection, we use a linear polarizer (LP, LPMIR050-MP2, Thorlabs, USA) at different angles (0$^{\circ }$, 45$^{\circ }$ and 90$^{\circ }$, see view A in Fig. 1). To prevent breakdown of the linear polarizer, only 40% of the radiation power is transmitted by the neutral density attenuator (AT, NDL-25S-4, Thorlabs, USA). For power measurements in orthogonal projections, a power meter (PM, 3A, Ophir, Israel) is used. To check the marginal consistency of SHG-FROG traces, we use an autocorrelator (PulseCheck, APE, Germany) and a Fourier spectrometer (OSA207C, Thorlabs, USA), as recommended by Ref.^41^. In total, the process of measuring all the data for one case took approximately 30 minutes. The measurement time for one SHG-FROG trace was approximately 7 minutes.

## Experimental results

By varying the settings of the PCs at the laser output and at the amplifier input and the pump power, we obtained a large number of regimes with different intensity profiles and durations, estimated from the intensity autocorrelator. Initially, we configured the PC at the laser output so that the isolator output has two pulses with elliptical orthogonal polarizations. Thus, the polarization at the isolator output already had a complex structure. Next, we varied the pump power and adjusted the PC at the amplifier input, evaluating the intensity autocorrelation in a single polarization projection (coinciding with the axis of the autocorrelator’s nonlinear crystal). First, we found the regime with the shortest intensity autocorrelation width of 133 fs with the total average power of 477 mW (pulse energy of 20 nJ) and measured the pulse electric field. Then, by varying the PC settings at the amplifier input and the amplifier pump power, we found a regime with the largest autocorrelation width of 641 fs and the total average power of 429 mW (pulse energy of 18 nJ) and repeated the electric field measurements. Therefore, we refer to the first measurement as the short-pulse measurement, and the second as the long-pulse measurement. Next, by adjusting the PC at the laser output, we ensured that the pulse was not separated into two and had a polarization close to the linear and low-energy sub-pulses. By adjusting the PC at the amplifier input and varying the pump power, we found the regime with the shortest intensity autocorrelation duration of 204 fs and measured the electric field vector. In terms of reconstruction error *G'*, defined as the root-mean-square error between measured and retrieved traces normalized to the measured trace area^44^, the first two measurements were quite stable, while in the third measured regime, the reconstruction errors of all SHG-FROG traces were at least twice as large. A large reconstruction error may indicate amplitude and phase instability in the pulse train^45^. Therefore, we refer to the first two measurements as the stable pulse train measurements and the third as the unstable pulse train measurement. In case of a stable pulse train, we present the results of the TURTLE reconstruction in the main text. For ease of reading, the measured and retrieved SHG-FROG traces, as well as the definition of the relative delay *τ* and phase *θ*, are given in Appendix A. The supplementary materials provide MATLAB scripts for plotting the reconstructed electric field and the Stokes parameters, azimuth angle, ellipticity angle, and Poincare-sphere trajectory for short and long pulses based on the corresponding data files. The supplementary materials also provide videos of the polarization ellipse rotation and electric field oscillations over time: the right shows the electric field projection ($E_x,E_y$), while the left shows the field oscillations in the $E_x$ projection over time. To understand the direction of polarization ellipse rotation, the video can be slowed down. In case of an unstable pulse train, we only consider the features of SHG-FROG trace reconstruction in three polarization projections and discuss the possibilities of instabilities.

For both stable and unstable pulse regimes, all measured SHG-FROG traces were symmetrical, indicating no change in the generation regime during the measurement of a single trace. Furthermore, for stable cases, the individual SHG-FROG traces were retrieved with relatively low reconstruction errors, and, most importantly, the independently measured 45° projection was well reproduced by the 45° FROG trace calculated from the two retrieved orthogonal components within the TURTLE procedure. This agreement indicates that the three measured projections were mutually consistent and that no significant change in the pulse state occurred during the acquisition of the stable regimes. In the unstable regime, the oscilloscope trace did not reveal a clear change of the laser regime, whereas the SHG-FROG reconstruction errors were approximately three times larger than in the stable cases. Therefore, the observed large reconstruction errors indicate instability of the output pulse train, but its exact origin cannot be unambiguously assigned to either the master oscillator or the amplifier.

### Stable pulse train

**Fig. 2:**
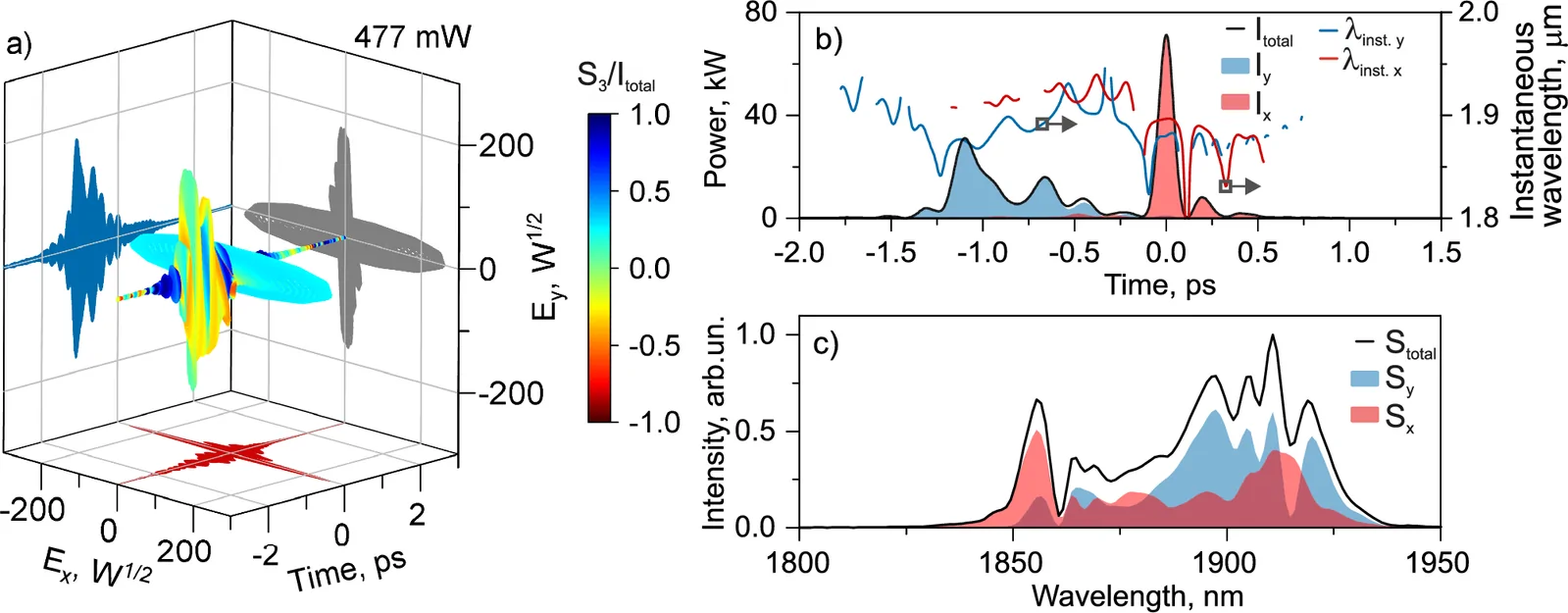
The electric field at the output of the amplifier in the short-pulse case (**a**), power in time (**b**), and spectral intensity (**c**) of horizontal and vertical projections are shown in red and blue fill, respectively. The black curve shows the total power in the time domain and intensity in the spectral domain. The instantaneous wavelength, calculated as $c/f_{inst}$, where c is the speed of light and $f_{inst}$ is the instantaneous frequency, for the horizontal and vertical polarization projections are shown in red and blue curves in figure (**b**). As the intensity in the polarization projections approaches zero, the instantaneous wavelength values become meaningless. The color in figure (**a**) denotes the normalized third Stokes parameter S$_{3}$/I$_{\textrm{total}}$, where 1 corresponds to left-hand circular polarization, 0 to linear polarization, and -1 to right-hand circular polarization.

The electric field for the case of a short pulse (Fig. 2a) shows that the wave packet has two temporally separated components with orthogonal, nearly linear polarization. This behavior is quantified by the instantaneous polarization extinction ratio (PER), defined as $10\log _{10}(P_{maj}/P_{min})$, where $P_{maj}$ and $P_{min}$ are the powers along the major and minor axes of the polarization ellipse at a certain value of time, respectively. The separation between the peaks of the orthogonal projections is 1099 fs, while the instantaneous PER reaches 17.7 dB and 27.3 dB at the respective maxima. In the temporal domain (Fig. 2b), the horizontal projection (red fill) shows a pulse with a duration of 94 fs and a peak power of 70 kW, accompanied by low-intensity sub-pulses. Meanwhile, in the vertical projection (blue fill), the pulse amplitude is modulated; there is a pulse with a duration of 197 fs and a peak power of 31 kW. As a result, the total peak power of 71 kW is determined primarily by the horizontal polarization component.

The instantaneous wavelengths of the orthogonal projections (Fig. 2b) differ significantly, so the values corresponding to the maximum intensity of the orthogonal projections differ by 21 nm. The main part of the pulse polarized in the horizontal projection is close to the transform limit, while the main part of the vertical projection exhibits frequency chirp. In the spectral domain (Fig. 2c), the spectral widths in orthogonal projections are 67 nm and 36 nm. The different instantaneous-wavelength profiles of the two orthogonal projections indicate different temporal phase evolutions of the *x*- and *y*-polarized field components. This produces a time-dependent relative phase between the components and, consequently, temporal evolution of the polarization ellipse and the Stokes parameters.

To calculate the average PER value, the average power of the polarization projection was plotted as a function of its angle. The ratio $10 \log _{10}(P_{av.max}/P_{av.min})$ was then calculated, where $P_{av.max}$ and $P_{av.min}$ are the maximum and minimum average powers. For the short-pulse case, the average PER is only 1.3 dB. A low value of this average PER indicates a low time-averaged linear-polarization contrast of the whole wave packet. However, it does not determine the instantaneous polarization state of the pulse, since circular or elliptical polarization requires information about both the instantaneous amplitude ratio and the relative phase between the orthogonal field components at the same time.

Since SHG-FROG and intensity autocorrelation measurements rely on a single scalar polarization projection, we analyze the variability of autocorrelation width and peak power with respect to the measured projection. Depending on the polarization projection, the intensity autocorrelation width varies from 131 fs to 495 fs and the peak power from 22 to 70 kW (see Supplementary Video sppia.mp4). Consequently, the range of estimated pulse durations based on the intensity autocorrelation width is from 87 to 333 fs. The peak power, obtained by dividing the total energy by the pulse duration estimated using the autocorrelation function, varies from 60 to 230 kW. Thus, the peak power may be overestimated by a factor of 3.3.

**Fig. 3:**
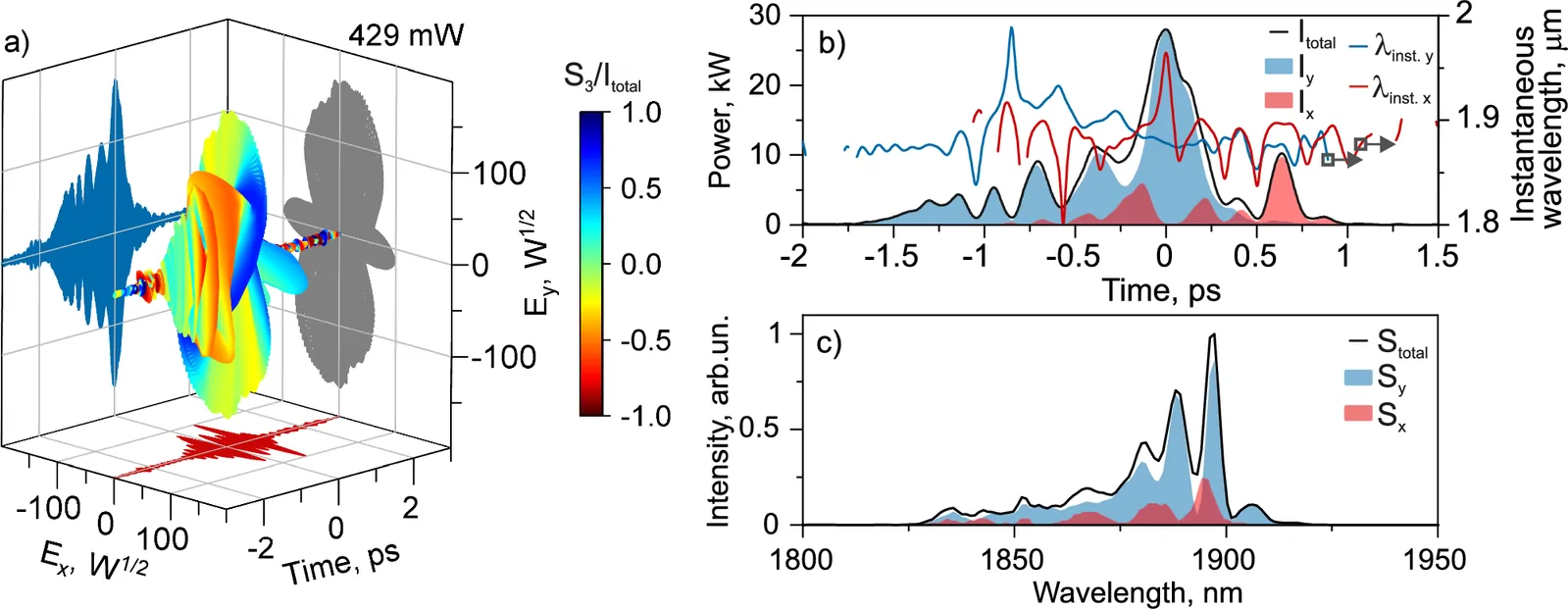
The electric field at the output of the amplifier in the long-pulse case (**a**), power in time (**b**), and spectral intensity (**c**) of horizontal and vertical polarization projections are shown in red fill and blue fill, respectively. The black curve shows the total power in the time domain and intensity in the spectral domain. The instantaneous wavelengths for the horizontal and vertical polarization projections are shown in red and blue curves in figure (**b**). The color in figure (**a**) denotes the normalized third Stokes parameter S$_{3}$/I$_{\textrm{total}}$, where 1 corresponds to left-hand circular polarization, 0 to linear polarization, and -1 to right-hand circular polarization.

The electric field for the long-pulse case (Fig. 3a) indicates that the polarization state becomes significantly closer to linear compared to the short-pulse regime. This trend is quantitatively confirmed by the polarization extinction ratio: the peak instantaneous PER reaches 21.6 dB, and the average PER increases to 5.8 dB.

Nevertheless, the intra-pulse polarization distribution is still complex, which is directly reflected in the temporal structure of the orthogonal field components (Fig. 3b). Both projections exhibit modulated temporal profiles, but with different durations. The pulse duration is 883 fs in the horizontal projection (red fill in Fig. 3b), and 270 fs in the vertical projection (blue fill in Fig. 3b). The total peak power of the wave packet is 27.8 kW, which coincides with the peak power in the vertical projection; for comparison, in the horizontal projection the peak power is 9.7 kW.

The instantaneous wavelength corresponding to the main peak of the horizontal projection (red curve in Fig. 3b) is constant, which means that the main peak is close to the transform limit, while the main peak of the vertical projection (blue curve in Fig. 3b) exhibits frequency chirp. The power spectral density is modulated in both projections (Fig. 3c), with spectral widths of 5.3 nm and 12.6 nm for the horizontal and vertical projections, respectively. This confirms that the observed temporal structure is accompanied by polarization-dependent spectral shaping.

Due to the complex electric field of the pulse, the pulse parameters in the long-pulse regime, as in the short-pulse case, depend strongly on the selected polarization projection. The intensity autocorrelation width varies from 220 fs to 1246 fs and the peak power from 8.9 kW to 27.8 kW (see Supplementary Video lppia.mp4). Therefore, determining the maximum peak power based on the intensity autocorrelation width and the energy of the wave packet may overestimate the peak power by 4.4 times.

The large differences in the spectra of orthogonal polarizations and their peak-like appearance in both cases are due to the complex nonlinear dynamics of the field vector in optical fibers, which is mainly due to the mutual action of dispersion and self-phase modulation; such dynamics in non-PM fibers are described by the coupled nonlinear Schrödinger equations^46^. Analyzing these dynamics requires numerical calculations, which consider both pulse parameters (amplitude-phase characteristics, polarization evolution, energy) and medium parameters (dispersion, nonlinearity, Raman response, losses, birefringence). Such a comprehensive theoretical study is beyond the scope of this experimental work. So, due to the nonlinear dynamics and fiber birefringence, the pulse electric field at the output of the non-PM thulium all-fiber MOPA has a complex structure, which depends on PCs settings. Therefore, measurements based on a single scalar projection (e.g., SHG-FROG or intensity autocorrelation) can lead to significant errors in the peak power and pulse duration estimation in non-PM all-fiber MOPA.

### Unstable pulse train

**Fig. 4:**
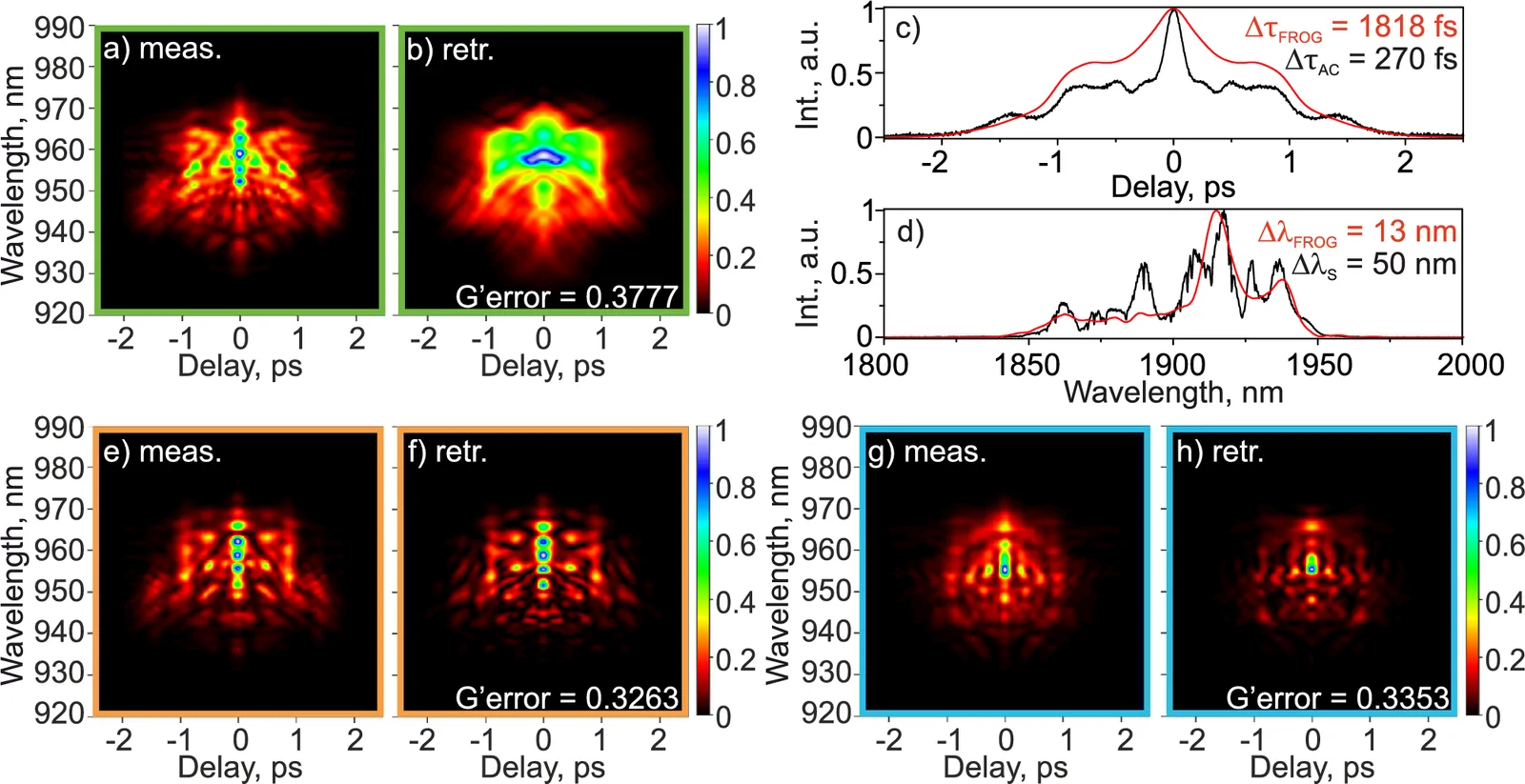
Measured and retrieved SHG-FROG traces for a 0$^\circ$-projection (**a**,**b**), a 45$^\circ$-projection (**e**,**f**), and a 90$^\circ$-projection (**g**,**h**) in the case of an unstable pulse train. Autocorrelation intensity trace measured by the autocorrelator and the spectral intensity measured by the Fourier spectrometer for 0$^\circ$-projection are shown in black in figures (**c**) and (**d**), respectively. The corresponding traces obtained after retrieving the SHG-FROG trace for 0$^\circ$-projection are shown in red.

With an average amplifier output power of 423 mW (pulse energy of 17.7 nJ) and single pulses at the amplifier input with predominantly linear polarization, the reconstructed SHG-FROG traces exhibit significant reconstruction errors (Fig. 4a-c), indicating instability of the pulse amplitude-phase characteristics. In contrast to the stable pulse-train regimes, a quantitative chirp analysis was not performed for the unstable pulse regime. SHG-FROG retrieval assumes that the measured trace corresponds to a reproducible pulse field. In the unstable regime, this assumption is not fulfilled: the measured SHG-FROG trace is averaged over a large number of pulses, while the pulse amplitude and phase may vary from pulse to pulse. Therefore, the retrieved temporal phase and instantaneous frequency should be regarded only as effective quantities associated with the averaged trace and may not correspond to the phase or chirp of any individual pulse in the train. For this reason, the FROG results, especially *G'* error, are used mainly as a diagnostic indicator of pulse-train instability^45^.

The SHG-FROG trace reconstruction error depends on the polarization projection. For the 0$^\circ$-projection, the reconstructed trace differs significantly from the measured one, while for the other two projections, a similar SHG-FROG trace structure is observed in the measured and reconstructed traces. The measured spectrum and autocorrelation for the 0$^\circ$-projection also differ from those obtained from the FROG trace retrieval (Fig. 4c,d). In autocorrelation measurements, pulse-train instability manifests itself as a pedestal in the autocorrelation function and a coherence peak^45^. The pedestal in the autocorrelation function is caused by variations in the pulse train, while the coherence peak is caused by the shortest repeating substructure in the pulses. However, the presence of a pedestal and a coherence peak does not guarantee pulse instability, since autocorrelation with these features may correspond to a stable case. For example, the autocorrelation function of a measured short pulse (see sppia.mp4 in the supplementary materials) at an angle of 126$^\circ$, has a pedestal (2.3 ps) and a peak (285 fs), although the autocorrelation function was calculated for the measured amplitude-phase characteristics of the pulse. Thus, using a reconstruction algorithm with *G'* error calculation allows one to identify instabilities in the pulse train.

Several mechanisms may contribute to the observed instability of the output pulse train. Firstly, instabilities may be observed in the master oscillator, although, judging by the oscilloscope trace, the pulse sequence corresponded to a stable generation regime during the measurement. This instability can be detected by measuring the FROG trace of the laser pulses. Second, instabilities associated with nonlinear effects may occur, such as modulation instability in the PM LMA fiber with anomalous dispersion or polarization instability in the active fiber with normal dispersion.

To more accurately determine the origin of the observed instability, we estimate the dispersion length, nonlinear length, soliton order, fission length for pulses with a duration of 175 fs estimated by the autocorrelation trace of unstable pulses; in this case, we will assume that all the energy (17.7 nJ) is contained in one soliton pulse, although in fact a significant part of the energy is contained in the pulse pedestal. Using the parameters of the PM LMA fiber ($\gamma = 0.26~1/(W\cdot km)$; $\beta _2=-79.6~ps^2/km$)^29^ and the pulse parameters ($P_p =156~kW, \tau _{0.5}=175~fs$), the nonlinear length ($L_{NL} =1/(\gamma ~P_0)$) is 2.4 cm, the dispersion length ($L_D=0.36\tau _{0.5}^{2}/|\beta _2|$) is 13.8 cm, the fundamental soliton order ($N=\sqrt{L_D/L_{NL}}$) is 2.4, the soliton fission length ($L_{fiss}\sim ~L_D/N$) is 5.8 cm^47^. The characteristic length of manifestation of modulation instability is 16 nonlinear lengths and is equal to 38 cm^48,49^. Since the soliton fission length is much shorter than the modulation instability length, the observed instability is unlikely to be related to modulation instability^48^. For reference, the B-integral of the part of the scheme after the laser output and before the active fiber is approximately 0.7 rad. For active fiber, it is approximately 28.2 rad; for PM LMA, it is approximately 7.7 rad. Such high values of the B-integral are typical for ultrafast fiber amplifiers and are due to the fact that in the active fiber there is a significant broadening of the spectrum, and in the PM LMA fiber there is nonlinear pulse compression^29,50,51^. However, the B-integral is not an accurate indicator of modulation instability.

Taking the value of the effective refractive index difference for the fast and slow axes of the active fiber to be $\Delta n = 1 \cdot 10^{-7}$ (as for SMF-28)^52,53^ and the nonlinear coefficient of the active fiber to be $8.85\cdot 10^{-3}~1/(W \cdot m)$^29^, the critical power of polarization instability in the active fiber is 56 W ($P_{cr}=3|\Delta n|\omega /(2\gamma ~c)$, where *ω = 992* rad/ps is the central angular frequency and c is the speed of light)^47^. According to previous calculations^29^, the peak power in the active fiber reaches approximately 2.5 kW (for 17.7 nJ pulse energy), which is higher than the critical power for polarization instability, so it can occur under certain pulse polarization conditions.

Thus, according to estimates, the most likely cause of the instability is the polarization instability effect. Despite these estimates, accurately identifying the cause of the instability is only possible through additional experiments and numerical modeling based on coupled Schrödinger equations using measured fiber parameters and the input pulse characteristics. Such a comprehensive study is beyond the scope of the present work.

## Discussion

Complex pulse polarization at the output of a non-PM all-fiber MOPA is possible not only with the special use of a fiber with high birefringence (PM LMA) but also, for example, in the case of multiple amplifier stages separated by isolators with unknown birefringence. The polarization of pulses passing through such isolators may change. Further propagation of pulses in the fiber may lead to an even greater change in the polarization state due to nonlinear polarization evolution^33,34^ and random birefringence in the fiber.

The main advantage of the TURTLE approach over standard scalar characterization methods is the reconstruction of the temporal evolution of the amplitude, phase, and polarization state of an ultrashort pulse. While conventional scalar measurements characterize only a selected polarization projection, the vector approach provides the two orthogonal complex field components, $E_x(t)$ and $E_y(t)$, together with their relative delay and phase. This makes it possible to calculate the total pulse intensity, Stokes parameters, polarization ellipse parameters, instantaneous PER, and the peak power of the complete vector pulse. When the polarization state significantly changes within the pulse envelope, these parameters may not be measured correctly using scalar methods alone.

The importance of such measurements is due to the complex polarization structure of ultrashort pulses generated by the developed non-PM MOPA. However, a simpler initial assessment of the pulse polarization at the output of such systems can be achieved by measuring the spectra and autocorrelation functions of the pulses for at least three pulse polarization projections. If the spectral shapes and autocorrelations of the pulses are preserved, it is most likely that the pulse polarization has a fairly uniform distribution across the pulse. If the shapes change significantly, an accurate assessment of the pulse parameters requires measuring the electric field vector of the pulse.

It is worth noting that PM thulium-doped fiber laser systems are generally stable sources that are more suitable for use outside of laboratories and typically have a high pulse PER value of about 20 dB, which makes the pulse polarization predictable, and vector measurements may not be necessary in this case. However, splicing dispersion-compensated PM fibers with standard PM fibers for laser or amplifier regime tuning may result in a significant PER degradation (up to 10 dB)^31^, which may require pulse electric field vector studies.

The accuracy of the reconstructed electric field vector is limited by the noise level of the SHG-FROG trace measurements. The reconstructed signal in the pulse wings becomes unreliable in low-intensity regions due to additive noise in the measured SHG-FROG trace^54^. In the present work, the estimated noise level of the SHG-FROG trace with the weakest polarization projection is on the order $10^{-2}$ of the maximum trace signal. Since the polarization parameters are calculated based on the reconstructed amplitude and phase characteristics, the reconstructed polarization structure is only relevant in the pulse sections where the power is more than 1% of the maximum total power, which corresponds to more than 710 W for the short pulse case and 270 W for the long pulse case. The corresponding measurement ranges are approximately from -1.4 ps to 0.5 ps for the short pulse case (Fig. 2b) and from -1.7 ps to 1 ps for the long pulse case (Fig. 3b). Although the robustness of TURTLE for reconstructing the relative delay and phase of orthogonal components under noise has been demonstrated previously^55^, a quantitative analysis of the polarization structure reconstruction in pulse wings as a function of noise is the subject of a separate study.

## Conclusion

In this work, we have experimentally demonstrated the measurement of the full electric field vector of sub-ps pulses at a wavelength of 1.9 µm generated by a non-PM thulium-doped all-fiber MOPA. Using the TURTLE method with SHG-FROG trace measurements, we reconstructed the amplitude, phase, and polarization of ultrashort pulses for stable and unstable pulse trains. For the stable pulse train, it has been shown that the pulse exhibits a complex time-dependent polarization structure, leading to strong dependence of the measured pulse duration, peak power, and autocorrelation trace on the selected polarization projection. Therefore, for pulses with complex electric fields, traditional scalar methods such as intensity autocorrelation or single-projection FROG can lead to significant errors in determining the peak power and pulse duration. In the regimes studied here, the peak power and pulse duration obtained from scalar measurements may be overestimated or underestimated by several times, depending on the selected polarization projection. We also demonstrated that for the unstable pulse train, large SHG-FROG reconstruction errors arise depending on the polarization projection. In this case, standard measurements of the spectrum and intensity autocorrelation are insufficient to reliably identify pulse instability, whereas vector measurements provide additional diagnostic capability. The obtained results highlight the importance of measuring the full electric field vector for accurate characterization of ultrashort pulses in non-PM fiber systems. A possible direction for further research is a detailed analysis of the influence of noise in the SHG-FROG trace on the pulse electric field vector characteristics.

## Supplementary Information

Supplementary Information.

## Figures and Tables

**Fig. 5:**
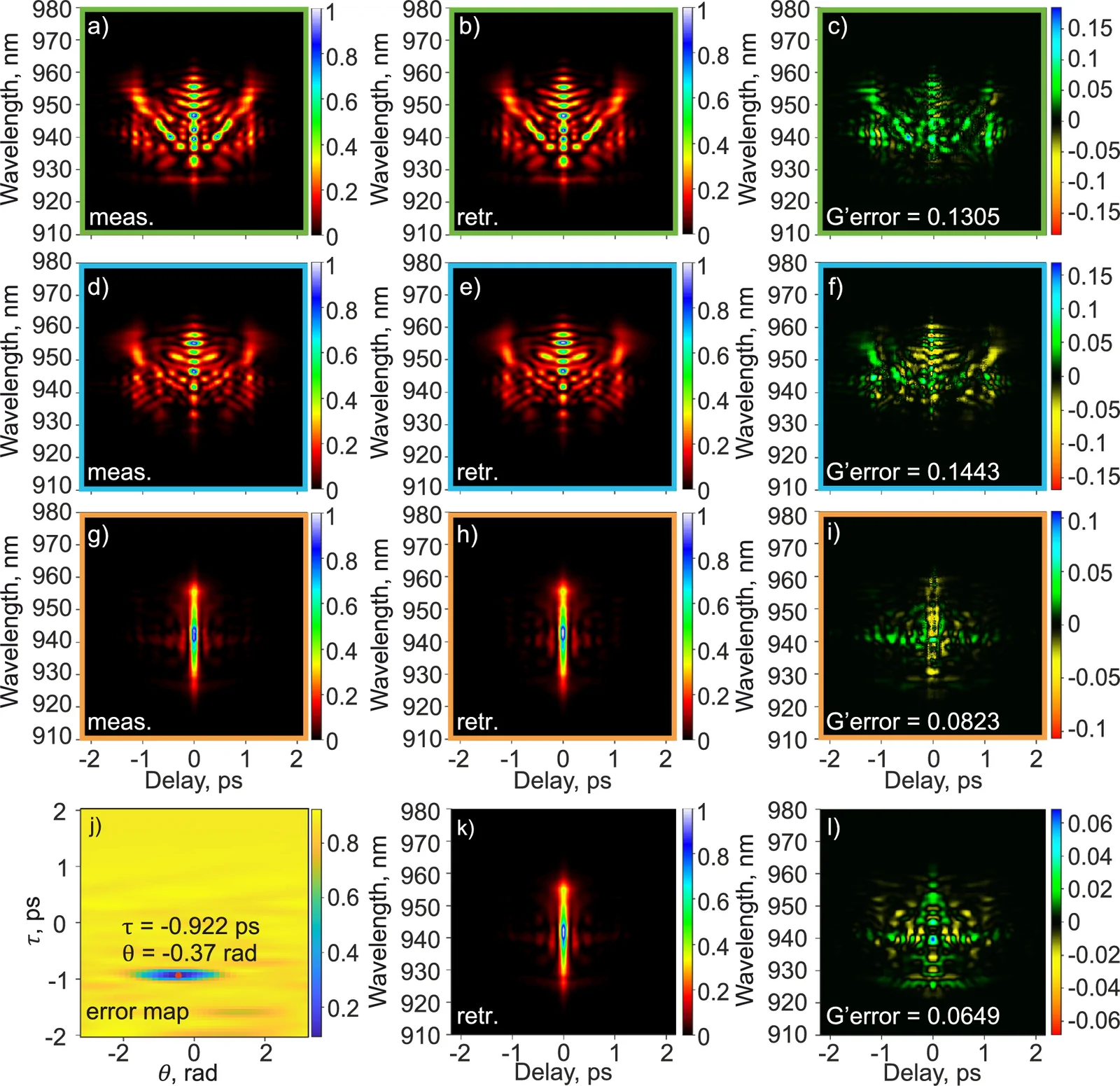
Measured and retrieved SHG-FROG traces and the difference between them for the $0^{\circ }$-projection (**a**-**c**), $90^{\circ }$-projection (**d**-**f**), and $45^{\circ }$-projection (**g**-**i**) for the short pulse case. *G'* error map between the retrieved 45-projection trace and the calculated trace obtained from orthogonal projections (**j**). The calculated trace obtained from orthogonal projections with minimum *G'* error (**k**). The difference between the (**h**) and (**k**) traces is (**l**). The red dot indicates the minimum in the *G'* error map.

**Fig. 6:**
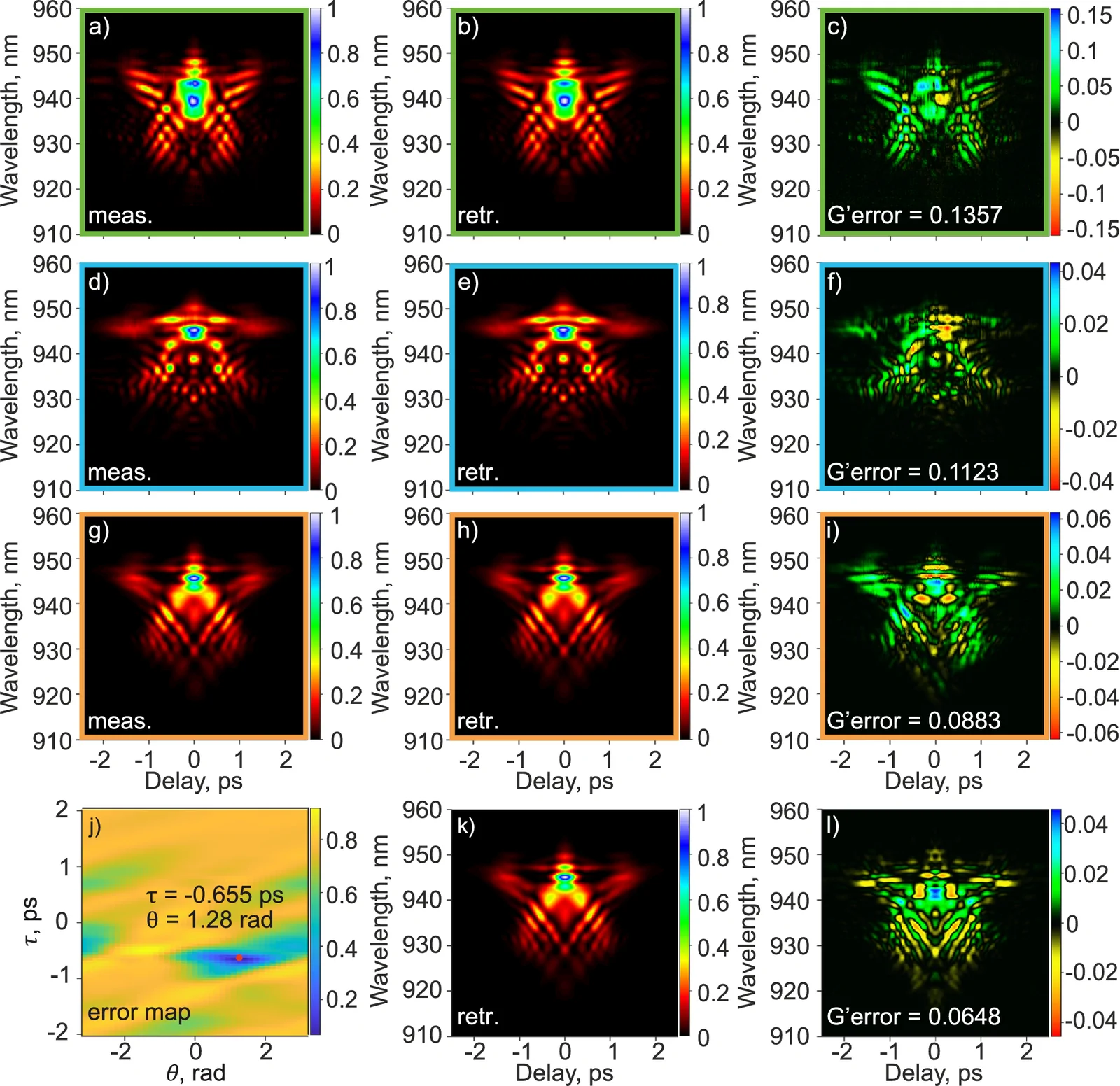
Measured and retrieved SHG-FROG traces and the difference between them for the $0^{\circ }$-projection (**a**–**c**), $90^{\circ }$-projection (**d**–**f**), and $45^{\circ }$-projection (**g**–**i**) for the long pulse case. *G'* error map between the retrieved 45-projection trace and the calculated trace obtained from orthogonal projections (**j**). The calculated trace obtained from orthogonal projections with minimum *G'* error (**k**). The difference between the (**h**) and (**k**) traces is (**l**). The red dot indicates the minimum in the *G'* error map.

## Data Availability

Data underlying the results presented in this paper is not publicly available at this time but may be obtained from the authors upon reasonable request.

## Appendix A: SHG-FROG traces and TURTLE retrievals

The SHG-FROG traces and error map for TURTLE retrieving for the short-pulse and the long-pulse cases are shown in Figs. 5 and 6, respectively. The measured and the retrieved SHG-FROG traces and the difference between them are shown for the 0$^\circ$-projection (a, b, c), for the 90$^\circ$-projection (d, e, f), and for the 45$^\circ$-projection (g, h, i). All measured FROG traces have the following parameters: *400× 701* points, delay resolution 10 fs, wavelength resolution 0.1 nm, delay range 4 ps, wavelength range 70 nm, and the center wavelength 945 nm. The retrieved FROG traces contain *4096× 4096* points, as we expand the grid in the measured FROG traces to obtain higher resolution in both time and spectral domains. So, the delay axis extends in the range of 20.488 ps, and the frequency axis is in the range from 218.5 to 418.4 THz, which corresponds to a wavelength range from 716.5 to 1371.7 nm.

For each case, we retrieve the amplitude and phase using the RANA approach, which reconstructs the pulse spectral intensity from the measured trace and employs a multigrid phase retrieval algorithm^44^. The reconstruction error *G'* values for all cases indicate excellent convergence. After reconstructing the pulse amplitude and phase in orthogonal projections, we calculate the SHG-FROG trace and the error *G'* between the reconstructed and measured SHG-FROG traces for a $45{^\circ }$-projection for different values of the relative delay *τ* and phase *θ*. To verify the existence of a pulse with an SHG-FROG trace corresponding to the measured $45^{\circ }$-projection trace, we also retrieve the measured trace for this projection, although this is not necessary. The *G'* error map for a short pulse case is shown in Fig 5j, and for a long pulse case, it is shown in Fig 6j. On the map, the delay values vary from − 2000 to 2000 fs with the step of 50 fs, and the phase values vary from − *π* to *π* rad with the step of *π /20* rad. The minimum point on the *G'* error map corresponds to the $(\tau , \theta )$ value, which we use as an initial guess for Nelder-Mead simplex two-parameter optimization. The minimum of this optimization corresponds to the values marked with a red dot and indicated on the error map. The FROG trace corresponding to the found minimum error *G'* for the short pulse case is shown in Fig. 5k, and for the long pulse case, in Fig. 6k. Finally, Figs. 5l and 6l show the difference between the calculated and reconstructed FROG traces for the $45{^\circ }$-projection for the short and long pulse cases, respectively.

We find the relative delay *τ* and phase *θ* with two relative time directions between orthogonal projections. For all TURTLE retrievals, for one of the time-direction choices, the calculated FROG trace patterns were dissimilar to those measured. This confirms that the relative time direction between the two polarization projections is correctly retrieved.
